# Measurement error of network clustering coefficients under randomly missing nodes

**DOI:** 10.1038/s41598-021-82367-1

**Published:** 2021-02-10

**Authors:** Kazuki Nakajima, Kazuyuki Shudo

**Affiliations:** Department of Mathematical and Computing Science, Tokyo Institute of Technology, Meguro-ku, Tokyo, 152-8552 Japan

**Keywords:** Computational science, Complex networks

## Abstract

The measurement error of the network topology caused by missing network data during the collection process is a major concern in analyzing collected network data. It is essential to clarify the error between the properties of an original network and the collected network to provide an accurate analysis of the entire topology. However, the measurement error of the clustering coefficient, which is a fundamental network property, has not been well understood particularly from an analytical perspective. Here we analytically and numerically investigate the measurement error of two types of clustering coefficients, namely, the global clustering coefficient and the network average clustering coefficient, of a network that is randomly missing some proportion of the nodes. First, we derive the expected error of the clustering coefficients of an incomplete network given a set of randomly missing nodes. We analytically show that (i) the global clustering coefficient of the incomplete network has little expected error and that (ii) conversely, the network average clustering coefficient of the incomplete network is underestimated with an expected error that is dependent on a property that is specific to the graph. Then, we verify the analytical claims through numerical simulations using three typical network models, i.e., the Erdős–Rényi model, the Watts–Strogatz model, and the Barabási–Albert model, and the 15 real-world network datasets consisting of five network types. Although the simulation results on the three typical network models suggest that the measurement error of the clustering coefficients on graphs with considerably small clustering coefficients may not behave like the analytical claims, we demonstrate that the simulation results on real-world networks that typically have enough high clustering coefficients sufficiently support our analytical claims. This study facilitates an analytical understanding of the measurement error in network properties due to missing graph data.

## Introduction

The characteristics of various real-world networks can be understood by measuring the topology of the corresponding graphs, with entities as nodes and their interactions as edges. One of the essential characteristics of real-world networks is that two nodes with a common neighbor are likely to be connected; this characteristic is captured by measuring the *clustering coefficients* of graphs. There are two types of clustering coefficients, namely, the global clustering coefficient (often referred to as the transitivity)^1,2^ and the network average clustering coefficient^3,4^. In a real-world network, both types of clustering coefficients are typically higher than those of a random graph with a similar number of nodes and edges^2,3^. The characteristics of high clustering coefficients have played essential roles in several areas of research, such as graph generative models^5–10^ and graph clustering algorithms^11,12^ for real-world networks.

The network data that are collected to measure the topology of a graph are often *incomplete* due to errors during the collection process. For example, in analyzing social networks through interviews with subjects^13^, some data may be invalid due to unanswered fields in a survey or divergent interpretations. When crawling in online social networks^14,15^ or web pages^16^, some graph data may be unavailable due to restricted access to the neighboring data of users^17^, dynamic changes in user interactions or web links, or bugs associated with public interfaces^18^. For topology measurements of the Internet^2,19,20^, a snapshot of the structure obtained by the union of a large set of paths taken by data packets that are sent between many different pairs may not contain computers with failed connections. Missing data during collection in real-world networks can be considered a general scenario in which some portion of the nodes or edges is missing from a graph.

The broad effects of missing network data on graph properties have long been studied^17,21–30^. In particular, *measurement errors* due to incomplete data between the properties of an original network and the collected network are a major concern in analyzing collected networks. For example, when researchers discuss the relative magnitude of the clustering coefficients of a collected network, underestimation and overestimation of the measured values can seriously affect the claims of the research. If such concerns are present, the qualitative effects of missing data, including overestimation or underestimation, can typically be predicted based on numerical simulations using certain real-world network data. Furthermore, the analytical investigation is essential to quantify measurement errors in general networks and to understand the network properties that cause those errors.

However, few results, particularly analytical results, regarding the measurement error of clustering coefficients caused by missing data have been clarified. Kossinets empirically claimed that the global clustering coefficient is only minimally affected by randomly missing nodes based on numerical simulations using scientific collaboration network data^27^; this claim has also been experimentally observed in other existing studies^28,29^. However, analytical results for general networks have not been obtained; it is not clear whether little measurement error against randomly missing nodes is the characteristic of the global clustering coefficient or results from the specific type and topology of real-world networks. Furthermore, neither analytical results nor empirical results regarding the network average clustering coefficient have yet been obtained.

In this paper, we analytically and numerically clarify the measurement error of the clustering coefficients of networks with *randomly missing nodes*. Although a scenario with a randomly missing nodes does not cover all possible scenarios of missing data that are encountered in the real world, it is a good starting point for analytically investigating the measurement errors of graph properties due to missing data. First, we theoretically investigate the clustering coefficients of an incomplete network in which some fraction of the nodes are randomly missing from a general undirected and unweighted graph. We approximate the expected relative errors of the clustering coefficients of an incomplete network given a set of randomly missing nodes. Our approximation decomposes the expectation of the clustering coefficients into a tractable product form. This is inspired by the concept of the mean-field approximation in statistical physics^31^ and was successfully applied in quantifying errors of graph properties caused by private nodes in social networks in our previous study^17^. The first analytical result shows that the global clustering coefficient of the incomplete network has little expected relative error, which supports the empirical claims made in previous studies^27–29^. The second analytical result claims that the network average clustering coefficient of an incomplete network is underestimated with an expected relative error that depends on a property that is specific to the graph. Finally, we verify the analytical claims through numerical simulations using the three typical network models, i.e., the Erdős–Rényi model^32^, the Watts–Strogatz model^3^, and the Barabási–Albert model^33^, and the 15 real-world network datasets consisting of five network types. Although the simulation results on the Erdős–Rényi model and the Barabási–Albert model suggest that the measurement errors of the clustering coefficients on graphs with considerably low clustering coefficients may not behave as shown in the analytical results, we demonstrate that our analytical claims sufficiently hold for real-world networks that typically have high clustering coefficients.

## Methods

### Definitions and notations

We represent a network as an undirected and unweighted graph with a set of *n* nodes, $V=\{v_1,\ldots , v_n\}$, and a set of edges, *E*. We ignore self-loops by convention^1,34–36^. We use $d_i$ to denote the degree of node $v_i$. We say that a triple of nodes $(v_j, v_i, v_k)$ is connected if $v_j$ is connected to $v_i$, $v_i$ is connected to $v_k$, and $j < k$. For a specific node $v_i$, the number of connected triples is $\frac{d_i(d_i-1)}{2}$. A triangle is defined as a connected triple $(v_j, v_i, v_k)$ in which $v_j$ and $v_k$ are connected. Let $\Delta _i = \{(v_j, v_k) \in E \mid (v_i, v_j) \in E \wedge (v_i, v_k) \in E \wedge j < k\}$ denote a set of two neighbors of node $v_i$ that are connected to each other. We use $t_i = |\Delta _i|$ to denote the number of triangles to which node $v_i$ belongs.

The global clustering coefficient^1,2^, denoted by *c*, is defined as the ratio of the total number of triangles to the total number of connected triples:

1 $$\begin{aligned} c = \frac{2 \sum _{v_i \in V} t_i}{\sum _{v_i \in V}d_i (d_i - 1)}, \end{aligned}$$

where a set of three nodes $\{v_j, v_i, v_k\}$ forms three different triangles. Next, the local clustering coefficient^3^ of node $v_i$, denoted by $c_i$, is defined as the ratio of the number of triangles to which $v_i$ belongs to the number of connected triplets to which $v_i$ belongs:

2 $$\begin{aligned} c_i = \frac{2 t_i}{d_i (d_i - 1)}, \end{aligned}$$

where we have $c_i = 0$ when $d_i = 0$ or $d_i = 1$. The network average clustering coefficient^3^, denoted by ${\overline{c}}$, is then defined as

3 $$\begin{aligned} {\overline{c}} = \frac{1}{n} \sum _{v_i \in V} c_i. \end{aligned}$$

We assume that an error at each node $v_i \in V$ can independently occur with probability $0 \le p \le 1$. We consider an error at node $v_i$ to result in the exclusion of node $v_i$ and its associated edges from *G*. We use $G' = (V', E')$ to represent the incomplete network obtained once nodes with errors and their edges have been excluded from *G*. Let $n'$ denote the number of nodes in $G'$, and let $d_i' = |\{v_j \mid (v_i, v_j) \in E'\}|$ denote the degree of node $v_i \in V'$ in $G'$. We use $t_i'$ to denote the number of triangles to which node $v_i \in V'$ belongs in $G'$.

According to Eq. (1), the global clustering coefficient of $G'$, denoted by $c'$, is defined as

4 $$\begin{aligned} c' = \frac{2 \sum _{v_i \in V'} t'_i}{\sum _{v_i \in V'}d_i' (d_i' - 1)}. \end{aligned}$$

Next, according to Eq. (2), the local clustering coefficient of node $v_i \in V'$, denoted by $c_i'$, is defined as

5 $$\begin{aligned} c_i' = \frac{2 t_i'}{d_i' (d_i' - 1)}, \end{aligned}$$

where we have $c_i' = 0$ when $d_i' = 0$ or $d_i' = 1$. According to Eq. (3), the network average clustering coefficient of $G'$, denoted by ${\overline{c}}'$, is then defined as

6 $$\begin{aligned} {\overline{c}}' = \frac{1}{n'} \sum _{v_i \in V'} c_i'. \end{aligned}$$

**Figure 1:**
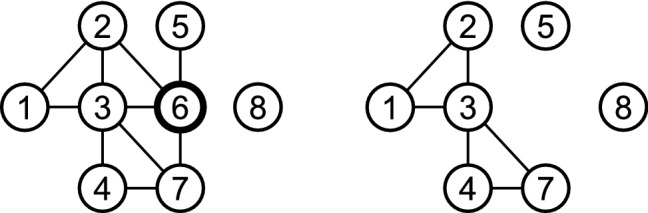
An example network (left) and the corresponding incomplete network (right) when node 6 is missing.

For example, let $G = (V, E)$ be the left graph in Fig. 1, where $v_i = i$ for $1 \le i \le 8$. For node 3 in *G*, we have $d_3 = 5$, $\Delta _3 = \{(1,2), (2,6), (4,7), (6, 7)\}$, $t_3 = 4$, and $c_3 = 0.4$. We also have $c = 0.469$ and ${\overline{c}} = 0.608$. Let the incomplete network $G'$ be the right graph in Fig. 1, which corresponds to the case in which node 6 is missing from *G*. For node 3 in $G'$, we have $d_3' = 4$, $t_3' = 2$, and $c_3' = 0.33$. We also have $c' = 0.5$ and ${\overline{c}}' = 0.524$.

### Data and Code

In our simulations, we first use nine synthetic graphs that were generated by using three typical network models: the Erdős–Rényi model (ER)^32^, the Watts–Strogatz model (WS)^3^, and the Barabási–Albert model (BA)^33^. For each $n = 1000$, $n = 5000$ and $n = 10,000$, the three synthetic graphs generated by the three models have the same number of nodes *n* and an average degree of 4. The reason we set the average degree to 4 is to clarify the effects of nodes with low degrees on the measurement error of the clustering coefficients. In the Watts–Strogatz model, we connect each node to 4 nearest neighbors in the ring topology so that a generated graph has an average degree of 4, and set the probability of rewiring each edge to 0.1 so that a generated graph has high clustering coefficients. In the Barabási–Albert model, we set the number of edges to attach from a new node to existing nodes to 2 so that a generated graph has an average degree of 4. Table 1 lists the numbers of nodes and edges and the clustering coefficients for the nine synthetic graphs used in our simulations. We note that the numbers of edges on the three synthetic graphs with 1000, 5000, and 10,000 nodes do not exactly match due to the characteristics of each model.

**Table 1:** Synthetic graphs generated by using Erdős–Rényi model (ER), Watts–Strogatz model (WS), and Barabási–Albert model (BA).

Name	|*V*|	|*E*|	*c*	${\overline{c}}$
ER1000	1000	1996	0.0054	0.0038
WS1000	1000	2000	0.1727	0.1983
BA1000	1000	1996	0.0119	0.0300
ER5000	5000	9954	0.0007	0.0005
WS5000	5000	10,000	0.3610	0.3800
BA5000	5000	9996	0.0022	0.0070
ER10000	10,000	20,116	0.0003	0.0002
WS10000	10,000	20,000	0.3540	0.3710
BA10000	10,000	19,996	0.0013	0.0031

We additionally use 15 publicly available datasets representing five types of real-world networks: a social network (SOC), a web graph (WEB), a computer network (COM), a co-authorship network (CA), and a co-purchasing network (CP). For simplicity, we obtain undirected, simple, connected graphs for all datasets by performing the following sequential preprocessing steps: (1) remove the directions of the edges if the original graph is directed, (2) treat multiple edges between the same pair of nodes as a single edge and delete loops, and (3) delete any nodes that are not contained in the largest connected component of the graph. These preprocessing steps do not affect our simulation results because they are performed before randomly removing nodes from the graph. Table 2 lists the network types, numbers of nodes and edges, and clustering coefficients for the 15 real-world network datasets used in our simulations. The source code and datasets used in our simulations are available^37^.

**Table 2:** Real-world network datasets.

Dataset	Type	|*V*|	|*E*|	*c*	${\overline{c}}$
Facebook^58^	SOC	63,392	816,886	0.148	0.222
Epinions^59^	SOC	75,877	405,739	0.066	0.138
YouTube^59^	SOC	1,134,890	2,987,624	0.006	0.081
CNR2000^58^	WEB	325,557	2,738,969	0.008	0.453
NotreDame^59^	WEB	325,729	1,090,108	0.088	0.235
Google^59^	WEB	855,802	4,291,352	0.055	0.519
Gnutella^58^	COM	62,561	147,878	0.004	0.005
CAIDA^58^	COM	190,914	607,610	0.061	0.158
Skitter^59^	COM	1,694,616	11,094,209	0.005	0.258
CiteSeer^58^	CA	227,320	814,134	0.456	0.676
DBLP^59^	CA	317,080	1,049,866	0.306	0.632
MathSciNet^58^	CA	332,689	820,644	0.137	0.410
Amazon0302^58^	CP	262,111	899,792	0.236	0.420
Amazon0601^58^	CP	403,364	2,443,311	0.166	0.418
Amazon0505^58^	CP	410,236	2,439,437	0.162	0.406

## Results

We provide the results regarding the measurement errors of the global clustering coefficient and the network average clustering coefficient of the incomplete network when each node on *G* is missing with probability *p*. We first present the analytical results and then we verify the analytical claims by conducting numerical simulations using three typical network models and the 15 real-world network datasets.

### Analytical results

We analytically investigate the error of the clustering coefficients of the incomplete network when each node in *G* is independently missing with probability *p*.

#### Global clustering coefficient

We focus on the expected relative error between *c* and $c'$ given a set of randomly missing nodes to quantify the measurement error of the global clustering coefficient. $E_{RN}[X]$ denotes the expected value of a random variable *X* given a set of randomly missing nodes in *G*, where *RN* is an abbreviation for “Randomly missing Nodes”. $1_{A}(x)$ denotes an indicator function for a set *A* that returns 1 if $x \in A$ and 0 otherwise. *Pr*[*B*] denotes the probability of an event *B*.

First, $d_i'$ follows the binomial distribution with parameters $d_i$ and $1-p$ given a set of randomly missing nodes because each neighbor of $v_i$ in *G* independently exists in $G'$ with probability $1-p$. Thus, for any node $v_i \in V$, we have

7 $$ E_{{RN}} [d_{i} ^{\prime } {\mid }v_{i}  \in V^{\prime}] = (1 - p)d_{i} , $$

8 $$ E_{{RN}} [(d_{i} ^{\prime } )^{2} n{\mid }v_{i}  \in V^{\prime}] = (1 - p)d_{i} [(1 - p)d_{i}  + p]. $$

Second, we derive $E_{RN}[t_i' \mid v_i \in V']$ for any node $v_i \in V$. For any two nodes $v_j \in V$ and $v_k \in V$, we define the random variable $X(j, k) = 1_{V'}(v_j) 1_{V'}(v_k)$. It holds that $t_i' = \sum _{(v_j, v_k) \in \Delta _i} X(j, k)$ under the condition that $v_i$ belongs to $V'$. We obtain the expectation of $t_i'$ given a set of randomly missing nodes under the condition that $v_i$ belongs to $V'$ as follows:

9 $$\begin{aligned} E_{RN}[t_i' \mid v_i \in V']&= \sum _{(v_j, v_k) \in \Delta _i} E_{RN}[X(j, k) \mid v_i \in V'] \end{aligned}$$

10 $$\begin{aligned}= \sum\limits_{{(v_{j} ,v_{k} ) \in \Delta _{i} }} P r[v_{j}  \in V^{\prime} \wedge v_{k}  \in V^{\prime}]  \end{aligned}$$

11 $$\begin{aligned}= \sum _{(v_j, v_k) \in \Delta _i} (1-p)^2 \end{aligned}$$

12 $$\begin{aligned}= (1-p)^2 t_i. \end{aligned}$$

Equation (9) holds true because of the linearity of the expected value. Equation (10) holds true because of the law of total expectation. Equation (11) holds true because $v_j$ and $v_k$ independently exist in $G'$ with probability $1-p$. Equation (12) holds true because of the definition of $t_i$.

Third, we derive the expectations of the numerator and denominator of $c'$: $E_{RN}[2 \sum _{v_i \in V'} t'_i]$ and $E_{RN}[\sum _{v_i \in V'}d_i' (d_i' - 1)]$. We define random variables $X_c(i) = t_i' 1_{V'}(v_i)$ and $Y_c(i) = d_i'(d_i'-1) 1_{V'}(v_i)$ for each node $v_i \in V$. Let $X_c = 2\sum _{v_i \in V'} t_i'$ and $Y_c = \sum _{v_i \in V'} d_i'(d_i'-1)$. It holds that $X_c = 2\sum _{v_i \in V} X_c(i)$ and $Y_c = \sum _{v_i \in V} Y_c(i)$. We obtain the expectation of $X_c$ given a set of randomly missing nodes as follows:

13 $$\begin{aligned} E_{RN}[X_c]&= 2\sum _{v_i \in V} E_{RN}[X_c(i)] \nonumber \\&= 2\sum _{v_i \in V} Pr[v_i \in V'] E_{RN}[t_i' \mid v_i \in V'] \nonumber \\&= 2(1-p)^3\sum _{v_i \in V} t_i. \end{aligned}$$

Equation (13) holds true because each node $v_i \in V$ independently exists in $G'$ with probability $1-p$ and Eq. (12) holds. Similarly, we obtain the expectation of $Y_c$ given a set of randomly missing nodes as follows:

14 $$\begin{aligned} E_{RN}[Y_c]&= \sum _{v_i \in V} E_{RN}[Y_c(i)] \nonumber \\&= \sum _{v_i \in V} Pr[v_i \in V'] E_{RN}[(d_i')^2 - d_i' \mid v_i \in V'] \nonumber \\&= \sum _{v_i \in V} (1-p) (E_{RN}[(d_i')^2 \mid v_i \in V'] - E_{RN}[d_i' \mid v_i \in V']) \nonumber \\&= (1-p)^3 \sum _{v_i \in V} d_i(d_i-1). \end{aligned}$$

Equation (14) holds because of Eqs. (7) and (8).

Finally, we approximate the expected value of $c'$ in Eq. (4) given a set of randomly missing nodes as a fraction of the expected value of the numerator and denominator by using Eqs. (13) and (14):

15 $$\begin{aligned} E_{RN}[c']&\approx \frac{E_{RN}[2 \sum _{v_i \in V'} t_i']}{E_{RN}[\sum _{v_i \in V'} d_i' (d_i' - 1)]} \nonumber \\&= c. \end{aligned}$$

This approximation is inspired by the concept of the mean-field approximation in statistical physics^31^ and was successfully applied in quantifying errors of graph properties caused by private nodes in social networks in our previous study^17^.

Equation (15) claims that the global clustering coefficient of $G'$ has little expected relative error given a set of randomly missing nodes, regardless of the probability *p*.

#### Network average clustering coefficient

We derive the expected relative error between ${\overline{c}}$ and ${\overline{c}}'$ given a set of randomly missing nodes. Let $$\left( {\begin{array}{c}a\\ b\end{array}}\right) $$ denote the binomial coefficient defined by a pair of integers $a \ge b \ge 0$, and let *m*! denote the factorial of a positive integer *m*.

First, we derive the expectation of $c_i'$ in Eq. (5) under the condition that $v_i$ belongs to $V'$: $E_{RN}[c_i' \mid v_i \in V']$. For node $v_i$ of degree $d_i = 0$ or $d_i = 1$, we have $E_{RN}[c_i' \mid v_i \in V'] = 0$. Now, we derive $E_{RN}[c_i' \mid v_i \in V']$ for node $v_i$ of degree $d_i \ge 2$. For any two nodes $v_j \in V$ and $v_k \in V$, we recall the random variable $X(j, k) = 1_{V'}(v_j) 1_{V'}(v_k)$. It holds that $t_i' = \sum _{(v_j, v_k) \in \Delta _i} X(j, k)$. Then, we have

16 $$\begin{aligned} E_{RN}[c_i' \mid v_i \in V']&= E_{RN}\left[ \frac{2\sum _{(v_j, v_k) \in \Delta _i} X(j, k)}{d_i'(d_i'-1)} \bigg | v_i \in V'\right] \nonumber \\&= 2\sum _{(v_j, v_k) \in \Delta _i} E_{RN}\left[ \frac{X(j, k)}{d_i'(d_i'-1)} \bigg | v_i \in V'\right] \nonumber \\&= 2\sum _{(v_j, v_k) \in \Delta _i} (1-p)^2 E_{RN}\left[ \frac{X(j, k)}{d_i'(d_i'-1)} \bigg | v_i \in V' \wedge v_j \in V' \wedge v_k \in V'\right] . \end{aligned}$$

Here, we have

17 $$\begin{aligned}&E_{RN}\left[ \frac{X(j, k)}{d_i'(d_i'-1)} \bigg | v_i \in V' \wedge v_j \in V' \wedge v_k \in V'\right] = E_{RN}\left[ \frac{1}{d_i'(d_i'-1)} \bigg | d_i' \ge 2\right] \end{aligned}$$

18 $$\begin{aligned} =&\sum _{k=2}^{d_i} {Pr[d_i' = k]\frac{1}{k(k-1)}} \nonumber \\ =&\sum _{k=2}^{d_i} \left( {\begin{array}{c}d_i-2\\ k-2\end{array}}\right) (1-p)^{k-2} p^{d_i-2 - (k-2)} \frac{1}{k(k-1)} \end{aligned}$$

19 $$\begin{aligned} =&\sum _{k=2}^{d_i} \frac{(d_i-2)!}{(d_i-k)! k!} (1-p)^{k-2} p^{d_i-k} \nonumber \\ =&\frac{1}{(1-p)^2 d_i(d_i-1)} \sum _{k=2}^{d_i} \left( {\begin{array}{c}d_i\\ k\end{array}}\right) (1-p)^k p^{d_i-k} \nonumber \\ =&\frac{1}{(1-p)^2 d_i(d_i-1)} [(1-p+p)^{d_i} - p^{d_i} - d_i(1-p)p^{d_i-1}] \end{aligned}$$

20 $$\begin{aligned} =&\frac{1}{(1-p)^2 d_i(d_i-1)} [1 - p^{d_i} - d_i(1-p)p^{d_i-1}]. \end{aligned}$$

Equation (17) holds true because $X(j,k) = 1$ and node $v_i$ has at least two neighbors $v_j$ and $v_k$ in $G'$ such that $v_j \in V'$ and $v_k \in V'$. Equation (18) holds true because the $d_i-2$ neighbors of $v_i$, excluding $v_j$ and $v_k$, independently exist in $G'$, each with probability $1-p$. Equation (19) holds true because $$\sum _{k=0}^{d_i} \left( {\begin{array}{c}d_i\\ k\end{array}}\right) (1-p)^k p^{d_i-k} = (1-p+p)^{d_i}$$ due to the binomial theorem, and the terms for $k = 0$ and $k = 1$ are subtracted from the total sum. Using Eqs. (16) and (20), we obtain $E_{RN}[c_i' \mid v_i \in V']$ for node $v_i$ of degree $d_i \ge 2$ as follows:

21 $$\begin{aligned} E_{RN}[c_i' \mid v_i \in V']&= 2\sum _{(v_j, v_k) \in \Delta _i} \frac{(1-p)^2 [1 - p^{d_i} - d_i(1-p)p^{d_i-1}]}{(1-p)^2 d_i(d_i-1)} \nonumber \\&= [1-p^{d_i}-d_i(1-p)p^{d_i-1}]c_i. \end{aligned}$$

We can incorporate $E_{RN}[c_i' \mid v_i \in V'] = 0$ for node $v_i$ of degree $d_i = 0$ or $d_i = 1$ into the equation $E_{RN}[c_i' \mid v_i \in V'] = [1-p^{d_i}-d_i(1-p)p^{d_i-1}]c_i$ for any degree $d_i \ge 0$ because $c_i = 0$ for $d_i =0$ or $d_i = 1$.

Then, we approximate the expectation of ${\overline{c}}'$ given a set of randomly missing nodes. Let $X_{{\overline{c}}} = \sum _{v_i \in V'} c_i'$. It holds that $X_{{\overline{c}}} = \sum _{v_i \in V} c_i' 1_{V'}(v_i)$ and $n' = \sum _{v_i \in V} 1_{V'}(v_i)$. First, we have the following equation by using Eq. (21):

22 $$\begin{aligned} E_{RN}[X_{{\overline{c}}}]&= \sum _{v_i \in V} (1-p) E_{RN}[c_i' \mid v_i \in V'] \nonumber \\&= (1-p) \sum _{v_i \in V} [1-p^{d_i}-d_i(1-p)p^{d_i-1}]c_i. \end{aligned}$$

We also have

23 $$\begin{aligned} E_{RN}[n']&= \sum _{v_i \in V} (1-p) E_{RN}[1_{V'}(v_i) \mid v_i \in V'] \nonumber \\&= \sum _{v_i \in V} (1-p) = (1-p)n. \end{aligned}$$

Finally, using Eqs. (22) and (23), the expectation of ${\overline{c}}'$ in Eq. (6) given a set of randomly missing nodes is approximated as follows:

24 $$\begin{aligned} E_{RN}[{\overline{c}}']&\approx \frac{E_{RN}[\sum _{v_i \in V'} c_i']}{E_{RN}[n']} \nonumber \\&= \tau _p {\overline{c}}, \end{aligned}$$

where the coefficient $\tau _p$ is defined as follows:

25 $$\begin{aligned} \tau _p = \frac{\sum _{v_i \in V} \left[ 1-p^{d_i}-d_i(1-p)p^{d_i-1}\right] c_i}{\sum _{v_i \in V} c_i}. \end{aligned}$$

Here, it holds that $1-p^{d_i}-d_i(1-p)p^{d_i-1} \le 1$ for any probability $0 \le p \le 1$ because $d_i \ge 0$ for each node $v_i$. Then, we have

26 $$\begin{aligned} 1 - \tau _p \ge 0 \end{aligned}$$

for any probability $0 \le p \le 1$.

Equation (24) and an inequality (26), it follows that the network average clustering coefficient of $G'$ is underestimated with an expected relative error $1-\tau _p$ given a set of randomly missing nodes.

### Simulation results

We verify our analytical results regarding the measurement error of the clustering coefficients of the incomplete networks with randomly missing nodes by conducting numerical simulations using the three typical network models and the 15 real-world network datasets. On each graph, each node and its associated edges are independently removed from the original graph with probability *p*. We set the probability *p* to values ranging from 0.0 to 0.9 in increments of 0.1. To estimate the true expected values of the clustering coefficients of the incomplete network when a fraction *p* of the nodes is randomly missing, we calculate the average values for 100 independent sets of randomly missing nodes for each probability *p*. We observe the average values along with the standard deviation across 100 independent sets of randomly missing nodes.

#### Global clustering coefficient

**Figure 2:**
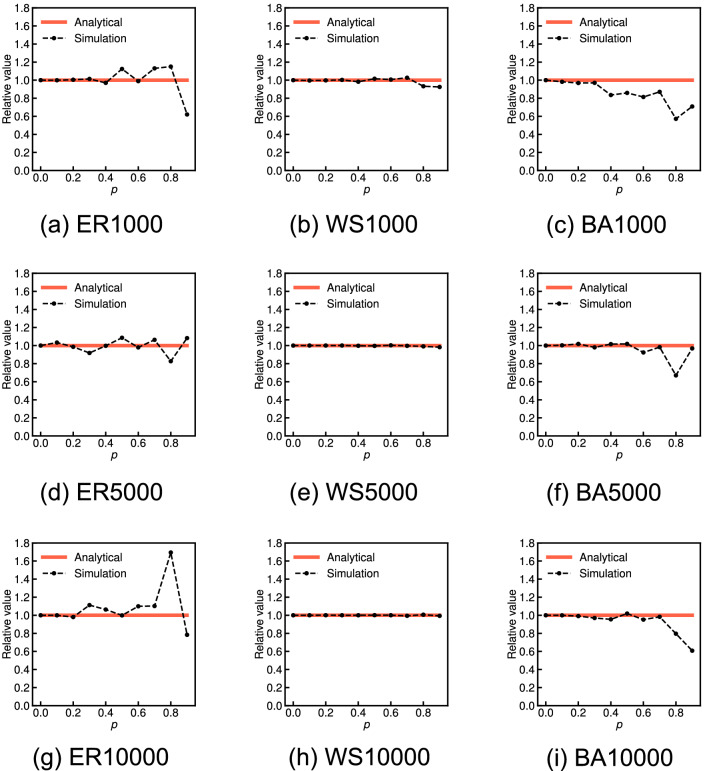
Comparison of the analytical and simulation results for the relative global clustering coefficient when each node is independently missing with probability *p* on ER, WS, and BA graphs for each 1000, 5000, and 10,000 nodes.

**Figure 3:**
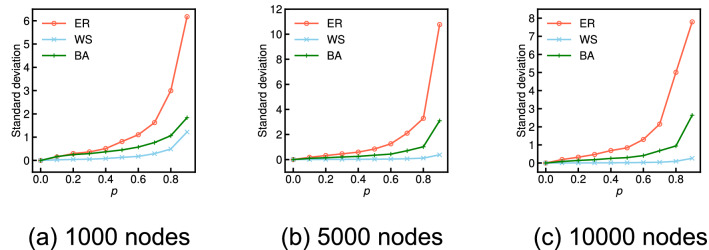
Comparison of the standard deviation of the relative global clustering coefficients across 100 independent sets of randomly missing nodes with the probability *p* on ER, WS, and BA graphs for each 1000, 5000, and 10,000 nodes.

Figure 2 shows the approximate expected values derived from Eq. (15) (red solid lines) and the average values over 100 independent simulations (black dashed lines) for various probabilities *p* on three network models for each $n = 1000$, $n = 5000$, and $n = 10,000$: the Erdős–Rényi model (ER), the Watts–Strogatz model (WS), and the Barabási–Albert model (BA). Both results are shown as the relative values with respect to the global clustering coefficient of the original graph. We observe that the global clustering coefficient of the incomplete network has little average relative error given a set of randomly missing nodes for all probabilities *p* of the WS graphs: this result sufficiently supports the analytical result. Conversely, the average relative values of the ER and BA graphs tend to greatly differ from the analytical result as the probability *p* increases. Figure 3 shows the standard deviation of the relative global clustering coefficients across 100 independent sets of randomly missing nodes for each probability *p* for the ER, WS, and BA graphs. We observe that the standard deviation in the ER and BA graphs tends to be considerably larger than that in the WS graphs as the probability *p* increases.

We consider that these differences in the relative errors and standard deviations between the ER and the BA and WS graphs arise from the magnitude of the original global clustering coefficient. In the ER and BA graphs, where the original global clustering coefficient is almost zero, we observed two extreme cases given a set of randomly missing nodes as the probability *p* increases: (i) All triangles are unfortunately destroyed and then the relative value is zero. (ii) Most triangles are not destroyed because of the limited number, but the degrees of most nodes are removed, and then the relative value is considerably larger than 1. Therefore, the measurement error of the global clustering coefficient given a random set of missing nodes can have a very large variance and greatly different from the analytical result for the ER and BA graphs. On the other hand, in the WS graphs, where the original global clustering coefficient is sufficiently high, such extreme cases hardly occur, and hence, the relative values on the WS graphs do not almost deviate from the analytical result.

**Figure 4:**
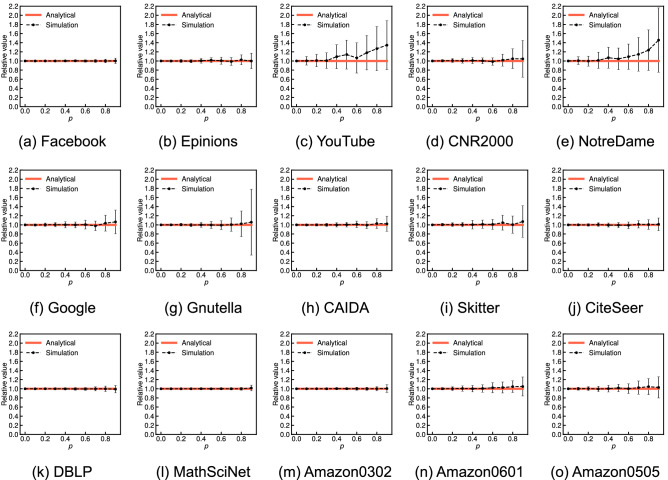
Comparison of the analytical and simulation results for the relative global clustering coefficient when each node is independently missing with probability *p* on 15 real-world network datasets. The error bar indicates the standard deviation across 100 independent sets of randomly missing nodes.

Then, Fig. 4 shows the approximate expected values derived from Eq. (15) (red solid lines) and the average values over 100 independent simulations (black dashed lines) for various probabilities *p* on the 15 real-world network datasets. The error bar indicates the standard deviation across 100 independent sets of randomly missing nodes. We have verified that the global clustering coefficient of the incomplete network has little average relative error given a set of randomly missing nodes for all datasets, except for YouTube and NotreDame, regardless of the type of network. These simulation results sufficiently support the analytical claim. We also observed that the standard deviations are small as in the simulation results on WS graphs, except for YouTube, NotreDame, and Gnutella. One possible reason for the large relative errors or standard deviations on YouTube, NotreDame, and Gnutella is that these three graphs have low global clustering coefficients compared with other datasets (0.006, 0.088, and 0.004, respectively). Here we recall that the relative errors were large on ER and BA graphs, which have almost zero global clustering coefficients. However, this reason may not be definitive due to small relative errors and standard deviations on Skitter, which has a low global clustering coefficient of 0.005. Comparing the simulation results on the 15 real-world network datasets in Fig. 4, we believe that the large relative errors or standard deviations on YouTube, NotreDame, and Gnutella are minor exceptions. We need to further investigate the factors underlying these differences in real-world network datasets in future work.

#### Network average clustering coefficient

**Figure 5:**
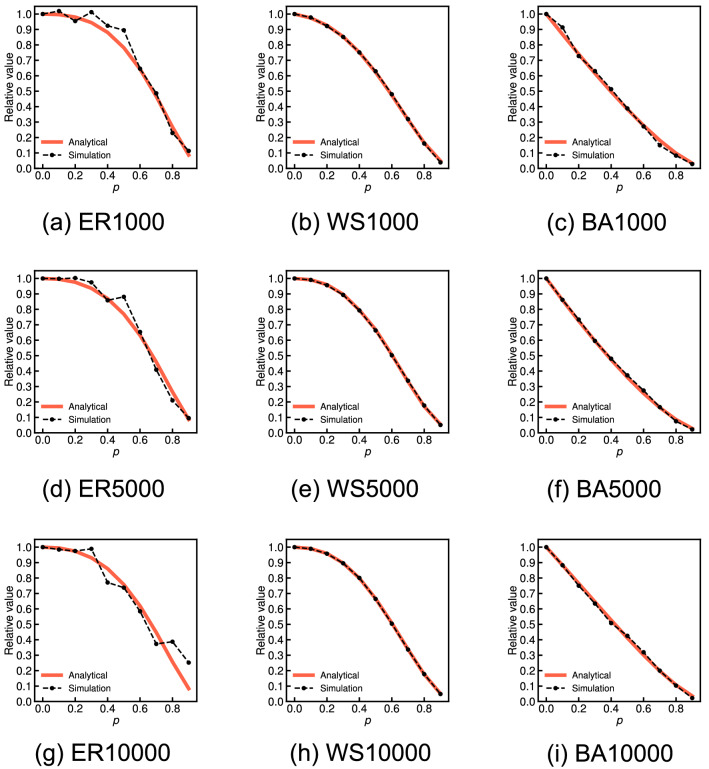
Comparison of the analytical and simulation results for the relative network average clustering coefficient when each node is independently missing with probability *p* on ER, WS, and BA graphs for each 1000, 5000, and 10,000 nodes.

**Figure 6:**
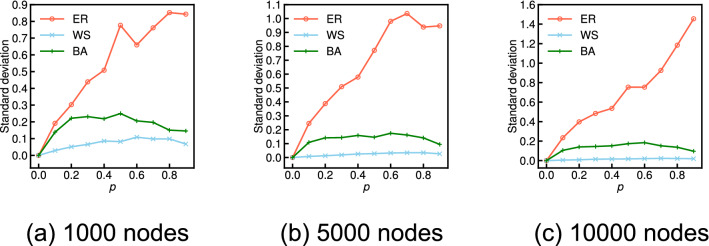
Comparison of the standard deviation of the relative network average clustering coefficients across 100 independent sets of randomly missing nodes with the probability *p* on ER, WS, and BA graphs for each 1000, 5000, and 10,000 nodes.

Figure 5 shows the approximate expected values derived from Eq. (24) (red solid lines) and the average values over 100 independent simulations (black dashed lines) for various probabilities *p* on the three network models (ER, WS, and BA graphs) for $n=$ 1000, 5000, and 10,000. We observe that the network average clustering coefficient is underestimated with an average relative error of $1-\tau _p$ on the WS graphs, which supports the analytical claim. Conversely, average relative errors have some errors compared with the analytical results for the ER and BA graphs. Figure 6 shows the standard deviation of the relative network average clustering coefficients across 100 independent sets of randomly missing nodes for each probability *p* on the ER, WS, and BA graphs. We observe the standard deviation in the WS graphs is clearly smaller than that in the ER and BA graphs. We consider that these differences in simulation results between the WS and the ER and BA graphs arise from the magnitude of the original network average clustering coefficient, as in the case of the global clustering coefficient.

**Figure 7:**
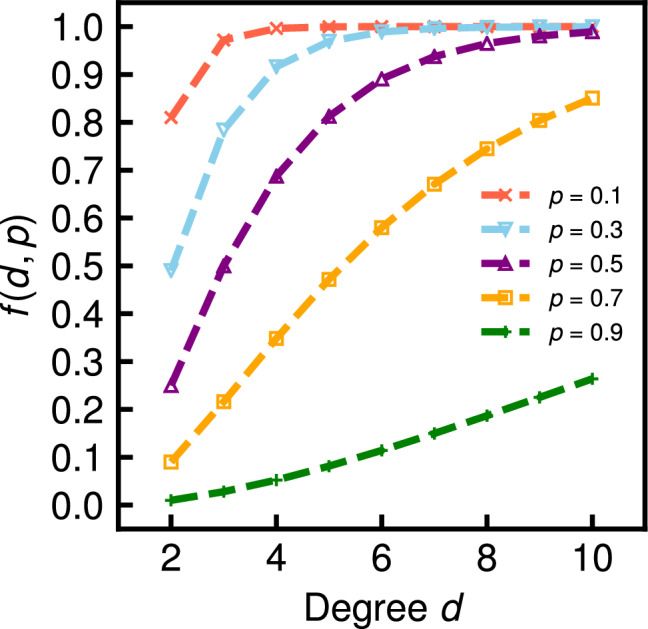
Function, $f(d, p) = 1-p^{d}-d(1-p)p^{d-1}$, for several degrees *d* and values of *p*.

We also find that the relative error in the BA graphs increases faster than the errors in the WS and ER graphs as the probability *p* increases. For example, when the probability *p* is 0.3, the relative errors on ER10000 and WS10000 are 0.08 and 0.10 (Fig. 5g,h), whereas that on BA10000 is 0.37 (Fig. 5i). The fast increase of the relative errors against the probability *p* caused by the degree distribution being biased to low degrees on the BA graphs in contrast to the ER and WS graphs. Here, we recall the definition of the coefficient $\tau _p$ in Eq. (25). The closer the quantity, $1-p^{d_i}-d_i(1-p)p^{d_i-1}$, for each node in the numerator is to 0, the more relative errors there are in the network average clustering coefficient of the incomplete network. Figure 7 shows the function, $f(d, p) = 1 - p^d - d(1-p)p^{d-1}$, for degrees $2 \le d \le 10$ and values $p = 0.1, 0.3, 0.5, 0.7$, and 0.9. We note that the function *f*(*d*, *p*) is not dependent on the node $v_i$ and the graph. We ignore the function values for $d = 0$ and 1 because $c_i = 0$ for nodes with $d_i = 0$ and 1. We see that as the degree *d* is smaller, the function value *f*(*d*, *p*) is lower for each value of *p*, i.e., the effect of nodes with the smaller degree *d* on the relative error is larger.

**Figure 8:**
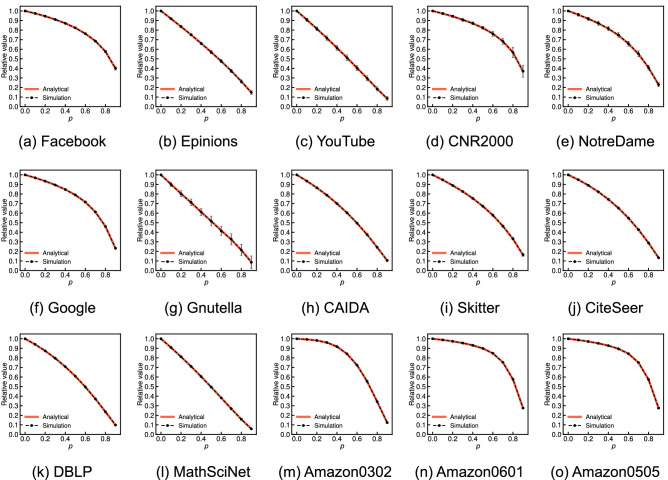
Comparison of the analytical and simulation results for the relative network average clustering coefficient when each node is independently missing with probability *p* on 15 real-world network datasets. The error bar indicates the standard deviation across 100 independent sets of randomly missing nodes.

Figure 8 shows the approximate expected values derived from Eq. (24) (red solid lines) and the average values over 100 independent simulations (black dashed lines) for various probabilities *p* on the 15 real-world network datasets. The error bar indicates the standard deviation across 100 independent sets of randomly missing nodes. We have verified that the network average clustering coefficient is underestimated with an average relative error of $$1-\tau _p$$ for all datasets regardless of the type of network, which sufficiently supports the analytical claim. We also found that the standard deviations are typically small as in the simulation results on the WS graphs. We further observe that the slope of the increase in the relative error of the network average clustering coefficient when the probability *p* increases is different depending on the real-world network. This difference results from the different proportions of nodes with a low degree in real-world networks, similar to the discussion in the case for the ER, WS, and BA graphs. Table 3 shows the cumulative degree distributions, *P*(degree $$\le d)$$, for $$d = $$2, 3, and 6 of 15 real-world network datasets. On YouTube, where nodes with degrees 6 or less account for 87.8% of the total, the increase in the relative errors of the network average clustering coefficient is considerably large (see Fig. 8c); e.g., the relative error is 0.489 if half of the nodes are removed. Conversely, on Amazon0601 and Amazon0505, where only approximately 20% of the nodes with degrees 6 or less, the slope of the relative error is relatively small (see Fig. 8n,o): the relative error is only 0.101 on Amazon0601 even if half of the nodes are removed.

**Table 3:** Cumulative degree distribution *P*(degree $\le d)$ for $d =$ 2, 3, and 6 of 15 real-world network datasets.

Dataset	*P*(degree $\le 2)$	*P*(degree $\le 3)$	*P*(degree $\le 6)$
Facebook	0.203	0.264	0.392
Epinions	0.620	0.696	0.802
YouTube	0.691	0.773	0.878
CNR2000	0.367	0.430	0.579
NotreDame	0.605	0.670	0.809
Google	0.288	0.374	0.551
Gnutella	0.606	0.670	0.739
CAIDA	0.442	0.566	0.763
Skitter	0.279	0.392	0.613
CiteSeer	0.290	0.439	0.692
DBLP	0.322	0.478	0.723
MathSciNet	0.492	0.628	0.808
Amazon0302	0.047	0.072	0.650
Amazon0601	0.068	0.109	0.214
Amazon0505	0.088	0.128	0.231

The final observation is that, in both the three network models and the 15 real-world network datasets, the analytical result for the network average clustering coefficient clearly provides a more accurate approximation than the case of the global clustering coefficient. We find that, for instance, the standard deviation of the relative network average clustering coefficients on the ER and BA graphs (see Fig. 6) is considerably smaller than that of the relative global clustering coefficients (see Fig. 3). We also see that the analytical results regarding the network average clustering coefficient on YouTube and NotreDame are almost the same as the simulation results (see Fig. 8c,e) in contrast to the case in the global clustering coefficient (see Fig. 4c,e). These differences empirically suggest that the measurement error in the network average clustering coefficient has little variance with respect to a set of randomly missing nodes. To fully explain the reason for these differences, it is necessary to analytically clarify the upper or lower bounds or the variance of the measurement errors of the clustering coefficients given a set of randomly missing nodes in future work.

## Discussion

We have studied the measurement error of two types of clustering coefficients, namely, the global clustering coefficient and the network average clustering coefficient, of a network with randomly missing nodes. First, we have analytically investigated the clustering coefficients of the incomplete network for a general undirected and unweighted graph. We have focused on the expected clustering coefficients given a set of randomly missing nodes to quantify the measurement errors. Then, to verify our analytical claims, we have numerically analyzed the measurement errors of the clustering coefficients on the three typical network models, i.e., the Erdős–Rényi model, the Watts–Strogatz model, and the Barabási–Albert model, and the 15 real-world network datasets consisting of five network types.

Our main results are as follows:

- In theory, the global clustering coefficient of the incomplete network shows little expected error given a set of randomly missing nodes.
- In theory, the network average clustering coefficient of the incomplete network is underestimated with an expected error that is dependent on $\tau _p$, which is a property specific to the graph, given a set of randomly missing nodes.
- The analytical results sufficiently hold for real-world networks that typically have high clustering coefficients, regardless of the network type. However, as the simulation results on the Erdős–Rényi model and the Barabási–Albert model suggest, the measurement errors of the clustering coefficients on graphs with considerably small clustering coefficients may not behave like those in the analytical results.
- The property $\tau _p$ can cause large measurement errors of the network average clustering coefficients on graphs with degree distributions that are biased toward low degrees.

Our results provide the following guidance for researchers investigating the triangular properties of collected networks. The global clustering coefficient provides reliable measurements under randomly missing nodes; even if a large percentage of nodes are randomly missing from the original network, the predicted measurement error is typically small in real-world scenarios. In contrast, researchers should carefully deal with the measured network average clustering coefficient when making claims based on the measurements. For example, if a researcher claims that the measured network average clustering coefficient is small, the claim may be overturned; the original value may be notably higher than the measurement.

Our study lefts future work of theoretically investigating the factors for the differences in the behaviors of measurement errors given a set of randomly missing nodes between the global clustering coefficient and the network average clustering coefficient. We empirically observed the analytical result for the network average clustering coefficient clearly provides a more accurate approximation than the case of the global clustering coefficient in both three typical network models and 15 real-world network datasets. We also empirically found that the measurement error of the network average clustering coefficient has a much smaller variance given a set of randomly missing nodes than the global clustering coefficient. Although we have only focused on the expected measurement errors of the clustering coefficients given a set of randomly missing nodes in this study, to fully explain these differences, it is also necessary to analytically clarify the upper or lower bounds or the variance of the measurement errors.

Our study also provides several directions for future research. First, we plan to study the measurement error caused by other types of missing data, such as the erroneous addition of nodes and the removal and addition of edges^25,27,30^. We believe that it is possible to analytically investigate the measurement error due to missing data under the assumption that nodes/edges are independently removed/added at random. Second, we would like to analytically clarify the measurement error of other graph properties. For example, there are extended clustering coefficients, such as the clustering coefficients in weighted^38^, directed^39^, or multiplex networks^40^; the network motifs^41^; and modified definitions of the clustering coefficients^36,42^. We consider that this study helps us to analytically study the measurement error due to missing graph data of particularly local graph properties, such as the triangular properties.

## Data Availability

The original real-world network datasets are publicly available: Facebook^43^, Epinions^44^, YouTube^45^, CNR2000^46^, NotreDame^47^, Google^48^, Gnutella^49^, CAIDA^50^, Skitter^51^, CiteSeer^52^, DBLP^53^, MathSciNet^54^, Amazon0302^55^, Amazon0601^56^, Amazon0505^57^.
